# TlPO_3_ and its comparison with isotypic RbPO_3_ and CsPO_3_

**DOI:** 10.1107/S2056989020011238

**Published:** 2020-08-21

**Authors:** Matthias Weil

**Affiliations:** a Institute for Chemical Technologies and Analytics, Division of Structural Chemistry, TU Wien, Getreidemarkt 9/164-SC, A-1060 Vienna, Austria

**Keywords:** crystal structure, isotypism, thallium, polyphosphate, structural similarity

## Abstract

The crystal structure of thallium(I) *catena*-polyphosphate, TlPO_3_, contains a polyphosphate chain extending parallel to [010] with a repeating unit of two phosphate tetra­hedra. The Tl^I^ atom is located in-between the polyphosphate chains and is bonded by oxygen atoms in a distorted [6 + 1] coordination in the form of a monocapped prism, with the longest Tl—O bond to the bridging O atom of the polyphosphate chain. A qu­anti­tative structural comparison with isotypic RbPO_3_ and CsPO_3_ reveals that the usually pronounced stereoactivity of the 6*s* ^2^ lone pair at the Tl^I^ atom is not apparent in the case of TlPO_3_.

## Abstract 2

TlPO_3_ is a *catena*-polyphosphate that represents the high-temperature polymorph of thallium(I) phosphate with a Tl:P ratio of 1:1.

## Chemical context   

The crystal chemistry of inorganic thallium(I) (or thallous) compounds is dominated by the presence of the 6*s* ^2^ electron lone pair that, in the majority of cases, is stereochemically active (Galy *et al.*, 1975 ▸). Therefore, crystal structures comprising a Tl^I^ atom mostly have unique structures, and isotypism with analogous phases where the Tl^I^ site is replaced by a metal cation of comparable size or by ammonium is comparatively rare. One of these cases pertains to the *catena*-polyphosphate series *M*PO_3_ (*M* = Tl, Rb, Cs) for which isotypism of TlPO_3_ with the alkali polyphosphates was reported on basis of indexed powder X-ray diffraction data (El Horr, 1991 ▸). Although single crystals were available, a refinement of the crystal structure was not performed at that time.

With the intention of obtaining detailed structure data for TlPO_3_ for a qu­anti­tative structural comparison with isotypic RbPO_3_ and CsPO_3_, single crystals of the thallium polyphos­phate phase were grown and the crystal structure refined using single-crystal X-ray data.

## Structural commentary   

**Figure 1:**
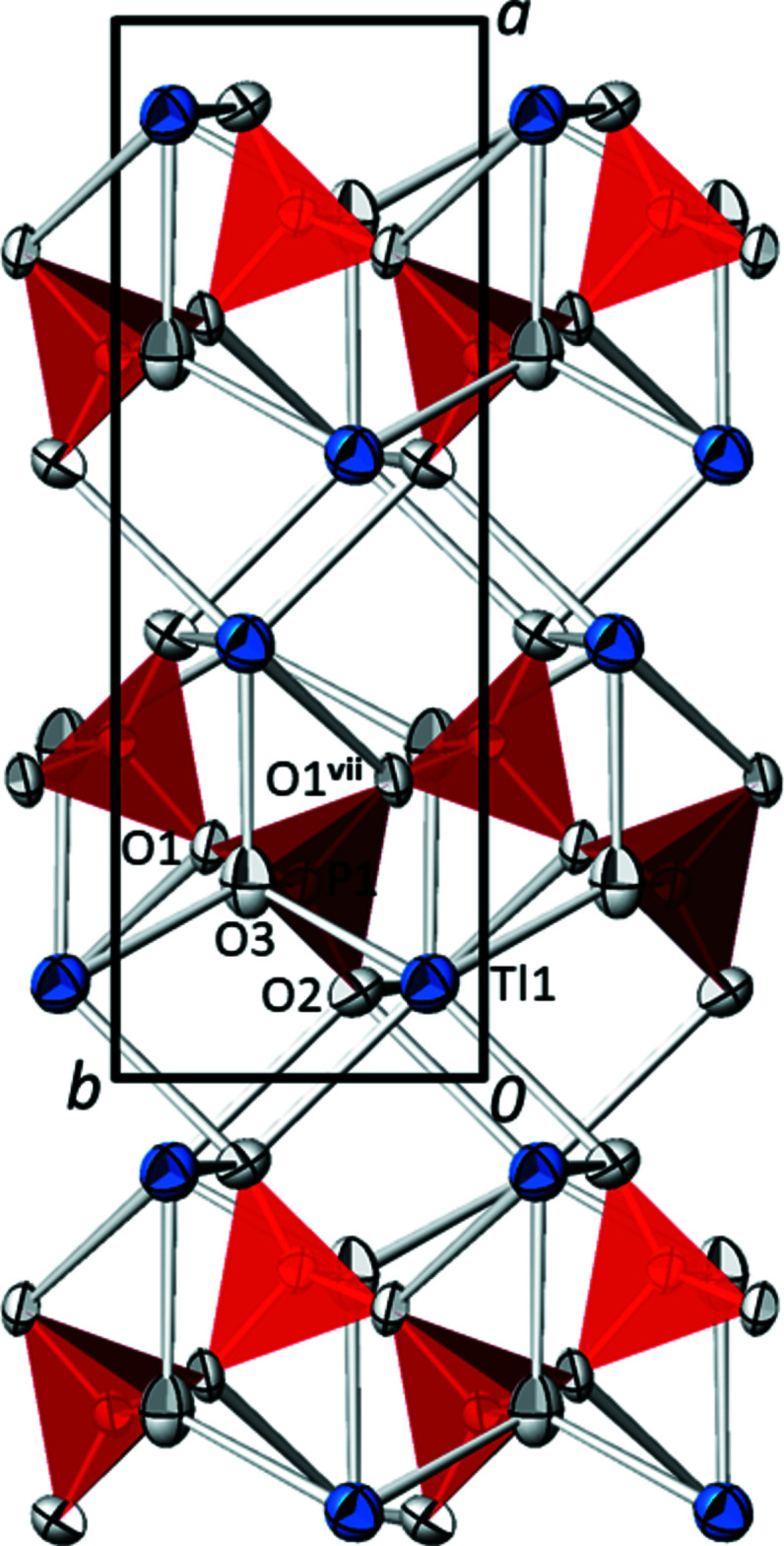
The crystal structure of TlPO_3_ in a projection along [00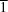]. Displacement ellipsoids are drawn at the 75% probability level. The symmetry code refers to Table 1 ▸.

**Figure 2:**
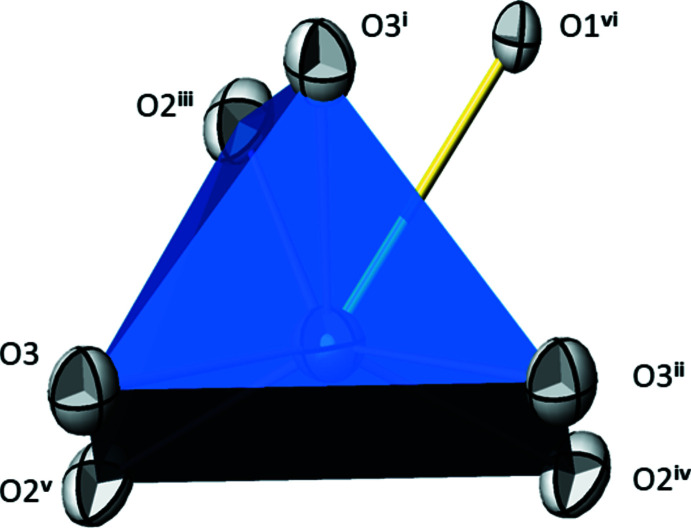
The [TlO_6 + 1_] monocapped prism in the crystal structure of TlPO_3_, with the long Tl—O bond shown in yellow. Displacement ellipsoids are drawn at the 75% probability level. Symmetry codes refer to Table 1 ▸.

The asymmetric unit of TlPO_3_ comprises one Tl, one P and three O sites, all on general positions. The crystal structure of TlPO_3_ is made up from a polyphosphate chain with a repeating unit of two phosphate tetra­hedra propagating along the [010] direction. Two polyphosphate chains with different orientations cross the unit cell (Fig. 1 ▸). The bond-length distribution (Table 1 ▸) within a PO_4_ tetra­hedron is characteristic of a polyphosphate chain (Durif, 1995 ▸), *i.e*. two long bonds to the bridging atoms O1 and O1^vii^ [mean 1.600 (8) Å; for symmetry code see Table 1 ▸] and two short bonds to the terminal O2 and O3 atoms [mean 1.483 (19) Å] are observed. The Tl^I^ atoms are situated between the chains and are coordinated to seven oxygen atoms. As has been done for thallium(I) oxoarsenates (Schroffenegger *et al.*, 2020 ▸), it is useful to classify the corresponding Tl^I^—O bonds into ‘short’ bonds less than 3.0 Å, and ‘long’ bonds greater than this threshold up to the maximum bond length of 3.50 Å for the first coordination sphere. The resulting [6 + 1] polyhedron can be derived from a monocapped trigonal prism where the capping O atom is that with the longest Tl—O bond (Fig. 2 ▸). This atom (O1) represents the bridging oxygen atom of the polyphosphate chain. Next to the Tl and two P atoms, atom O1 has no further coordination partners. The terminal O2 and O3 atoms of the polyphosphate chain each are bonded to one P and to three Tl atoms in the form of a distorted tetra­hedron.

Bond-valence-sum (BVS) calculations (Brown, 2002 ▸) for TlPO_3_ were carried out with the values provided by Locock & Burns (2009 ▸) for Tl^I^—O bonds, and by Brown & Altermatt (1985 ▸) for P—O bonds. The obtained BVS values (in valence units) of 0.88 for Tl1, 4.97 for P1, 2.17 for O1, 1.97 for O2 and 1.82 for O3 differ somewhat from the idealized values for atoms with a formal charge of +1, +5 and −2, respectively. In consequence, the global instability index *GII* (Salinas-Sanchez *et al.*, 1992 ▸) of 0.14 valence units is rather high and indicates a stable but strained structure (*GII* values < 0.1 valence units are typical for unstrained structures, *GII* values between 0.1 and 0.2 are characteristic of structures with lattice-induced strain, and *GII* values > 0.2 indicate unstable structures).

Apparently, the usually observed stereochemical activity of the 6*s* ^2^ electron lone pair at the Tl^I^ atom is not very pronounced in the case of TlPO_3_, and a crystal structure isotypic with those of the room-temperature forms of RbPO_3_ and CsPO_3_ is realized (Table 1 ▸). This may be due to the comparable ionic radii for monovalent Tl, Rb^+^ and Cs^+^ cations of 1.50, 1.52 and 1.67 Å, respectively, using a coordination number of six (Shannon, 1976 ▸; values for a coordination number of seven were not listed for Tl and Cs).

Whereas the isotypic polyphosphates RbPO_3_ and CsPO_3_ show structural phase transitions to two (Holst *et al.*, 1994 ▸) and to one high-temperature phases (Chudinova *et al.*, 1989 ▸), a structural phase transition at higher temperatures has not been reported for TlPO_3_. On the contrary, the tetra­metaphosphate Tl_4_P_4_O_12_ converts at 690 K to the title polyphosphate (Dostál *et al.*, 1969 ▸) that therefore represents the high-temperature form of a phosphate with a Tl:P ratio of 1:1.

For a qu­anti­tative structural comparison of the three isotypic *M*PO_3_ (*M* = Tl, Rb, Cs) structures, the program *compstru* (de la Flor *et al.*, 2016 ▸) available at the Bilbao Crystallographic Server (Aroyo *et al.*, 2006 ▸) was used. Numerical details of the comparison with the TlPO_3_ structure as the reference are collated in Table 2 ▸. The low values for the degree of lattice distortion (*S*), the similarity index Δ and the arithmetic mean distance of paired atoms (*d*_av_) indicate very similar structures, with the highest absolute displacement of atom O3 in each case.

## Synthesis and crystallization   

A mass of 0.50 g Tl_2_CO_3_ was immersed in 3 ml of concentrated phospho­ric acid (85%_wt_) in a glass carbon crucible. The mixture was heated within six hours to 573 K, kept at that temperature for ten hours and slowly cooled to room temperature over the course of twelve hours. The obtained highly viscous phosphate flux was leached with a mixture of water and methanol (*v*/*v* = 1:4). After separation of the liquid phase through suction filtration, colourless crystals of TlPO_3_, mostly with a platy form, were obtained.

## Refinement   

Crystal data, data collection and structure refinement details are summarized in Table 3 ▸.

The lattice parameters determined in the current study are in good agreement with those of the previous report [*a* = 12.270 (7), *b* = 4.263 (2), *c* = 6.328 (4) Å, *β* = 96.72 (3)°, *V* = 328.7 Å^3^; El Horr, 1991 ▸], however with higher precision.

For better comparison with the two isotypic structures of RbPO_3_ and CsPO_3_, the setting of the unit cell (cell choice 2 of space group No. 14), starting coordinates and atom labelling were adapted from RbPO_3_ (Corbridge, 1956 ▸). The maximum and minimum electron densities in the final difference-Fourier synthesis are located 0.65 and 0.74 Å, respectively, from the Tl1 site.

## Supplementary Material

Crystal structure: contains datablock(s) I, global. DOI: 10.1107/S2056989020011238/hb7938sup1.cif

Structure factors: contains datablock(s) I. DOI: 10.1107/S2056989020011238/hb7938Isup2.hkl

CCDC reference: 2023579

Additional supporting information:  crystallographic information; 3D view; checkCIF report

## Figures and Tables

**Table 1:** Comparison of bond lengths in the isotypic *M*PO_3_ polyphosphates (*M* = Tl, Rb, Cs) (*a*) This work; (*b*) *a* = 12.123 (2), *b* = 4.228 (2), *c* = 6.479 (2) Å, *β* = 96.33 (33)° (Corbridge, 1956 ▸); (*c*) *a* = 12.6162 (11), *b* = 4.2932 (4), *c* = 6.7575 (6) Å, *β* = 96.068 (5)° (Weil & Stöger, 2020 ▸).

	*M* = Tl*^*a*^*	*M* = Rb*^*b*^*	*M* = Cs*^*c*^*
*M*—O3^i^	2.867 (5)	2.920	3.1097 (18)
*M*—O3	2.889 (4)	2.971	3.0980 (14)
*M*—O3^ii^	2.935 (4)	2.948	3.0983 (14)
*M*—O2^iii^	2.943 (5)	2.973	3.0981 (14)
*M*—O2^iv^	2.963 (4)	2.905	3.0431 (15)
*M*—O2^v^	2.997 (4)	3.024	3.1455 (15)
*M*—O1^vi^	3.216 (4)	3.196	3.3649 (14)
P—O2	1.469 (5)	1.474	1.4793 (16)
P—O3	1.497(5	1.438	1.4919 (16)
P—O1	1.595 (4)	1.621	1.6134 (16)
P—O1^vii^	1.606 (4)	1.624	1.6183 (16)
			

Symmetry codes (i) −*x* + 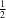, *y* − 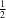, −*z* + 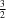; (ii) *x*, *y* − 1, *z*; (iii) *x*, *y*, *z* + 1; (iv) −*x*, −*y*, −*z* + 1; (v) −*x*, −*y* + 1, −*z* + 1; (vi) *x*, *y* − 1, *z* + 1; (vii) −*x* + 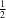, *y* − 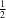, −*z* + 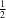.

**Table 2:** Absolute atomic displacements (Å) of isotypic RbPO_3_ and CsPO_3_ relative to TlPO_3_, as well as lattice distortion (*S*), arithmetic mean distance *d*_av_ (Å) and measure of similarity (Δ)

	Rb	Cs
*M*1	0.0656	0.0703
P1	0.0505	0.0595
O1	0.0421	0.0494
O2	0.0996	0.0735
O3	0.1332	0.1793
S	0.0099	0.0245
*d*_av_	0.0782	0.0864
Δ	0.060	0.039

**Table 3:** Experimental details

Crystal data	
Chemical formula	TlPO_3_
*M*_r_	283.34
Crystal system, space group	Monoclinic, *P*2_1_/*n*
Temperature (K)	296
*a*, *b*, *c* (Å)	12.2315 (12), 4.2432 (7), 6.3039 (1)
β (°)	96.727 (7)
*V* (Å^3^)	324.92 (6)
*Z*	4
Radiation type	Mo *K*α
μ (mm^−1^)	49.99
Crystal size (mm)	0.09 × 0.08 × 0.01

Data collection	
Diffractometer	Bruker APEXII CCD
Absorption correction	Numerical (*HABITUS*; Herrendorf, 1997 ▸)
*T*_min_, *T*_max_	0.110, 0.536
No. of measured, independent and observed [*I* > 2σ(*I*)] reflections	6570, 1189, 912
*R*_int_	0.059
(sin θ/λ)_max_ (Å^−1^)	0.758

Refinement	
*R*[*F* ^2^ > 2σ(*F* ^2^)], *wR*(*F* ^2^), *S*	0.027, 0.054, 0.99
No. of reflections	1189
No. of parameters	46
Δρ_max_, Δρ_min_ (e Å^−3^)	1.85, −1.64

Structure solution: isomorphous replacement. Computer programs: *APEX3* and *SAINT* (Bruker, 2018 ▸), *SHELXL2018/3* (Sheldrick, 2015 ▸), *ATOMS* (Dowty, 2006 ▸) and *publCIF* (Westrip, 2010 ▸).

## supplementary crystallographic information

### Crystal data

**Table:**

TlPO_3_	*F*(000) = 480
*M**_r_* = 283.34	*D*_x_ = 5.792 Mg m^−^^3^
Monoclinic, *P*2_1_/*n*	Mo *K*α radiation, λ = 0.71073 Å
*a* = 12.2315 (12) Å	Cell parameters from 2009 reflections
*b* = 4.2432 (7) Å	θ = 3.4–29.2°
*c* = 6.3039 (1) Å	µ = 49.99 mm^−^^1^
β = 96.727 (7)°	*T* = 296 K
*V* = 324.92 (6) Å^3^	Plate, colourless
*Z* = 4	0.09 × 0.08 × 0.01 mm

### Data collection

**Table:**

Bruker APEXII CCD diffractometer	912 reflections with *I* > 2σ(*I*)
Radiation source: fine-sealed tube	*R*_int_ = 0.059
ω– and φ–scans	θ_max_ = 32.6°, θ_min_ = 3.4°
Absorption correction: numerical (*HABITUS*; Herrendorf, 1997)	*h* = −18→18
*T*_min_ = 0.110, *T*_max_ = 0.536	*k* = −6→6
6570 measured reflections	*l* = −9→9
1189 independent reflections	

### Refinement

**Table:**

Refinement on *F*^2^	46 parameters
Least-squares matrix: full	0 restraints
*R*[*F*^2^ > 2σ(*F*^2^)] = 0.027	*w* = 1/[σ^2^(*F*_o_^2^) + (0.0219*P*)^2^] where *P* = (*F*_o_^2^ + 2*F*_c_^2^)/3
*wR*(*F*^2^) = 0.054	(Δ/σ)_max_ < 0.001
*S* = 0.99	Δρ_max_ = 1.85 e Å^−^^3^
1189 reflections	Δρ_min_ = −1.64 e Å^−^^3^

### Special details

**Table:**

Geometry. All esds (except the esd in the dihedral angle between two l.s. planes) are estimated using the full covariance matrix. The cell esds are taken into account individually in the estimation of esds in distances, angles and torsion angles; correlations between esds in cell parameters are only used when they are defined by crystal symmetry. An approximate (isotropic) treatment of cell esds is used for estimating esds involving l.s. planes.

### Fractional atomic coordinates and isotropic or equivalent isotropic displacement parameters (Å^2^)

**Table:**

	*x*	*y*	*z*	*U*_iso_*/*U*_eq_	
Tl1	0.08906 (2)	0.14776 (5)	0.77279 (4)	0.02917 (9)	
P1	0.18222 (13)	0.4944 (3)	0.3224 (2)	0.0183 (3)	
O1	0.2184 (4)	0.7447 (9)	0.1551 (6)	0.0202 (9)	
O2	0.0814 (4)	0.3466 (9)	0.2180 (8)	0.0331 (11)	
O3	0.1858 (4)	0.6404 (9)	0.5393 (7)	0.0316 (11)	

### Atomic displacement parameters (Å^2^)

**Table:**

	*U*^11^	*U*^22^	*U*^33^	*U*^12^	*U*^13^	*U*^23^
Tl1	0.02974 (15)	0.02897 (13)	0.02893 (13)	−0.00135 (13)	0.00402 (10)	0.00170 (11)
P1	0.0196 (8)	0.0134 (6)	0.0227 (7)	−0.0015 (6)	0.0060 (6)	−0.0018 (5)
O1	0.024 (2)	0.0119 (15)	0.025 (2)	−0.0029 (16)	0.0039 (18)	0.0000 (15)
O2	0.021 (2)	0.026 (2)	0.052 (3)	−0.004 (2)	0.002 (2)	−0.005 (2)
O3	0.046 (3)	0.025 (2)	0.026 (2)	0.001 (2)	0.011 (2)	−0.0035 (18)

### Geometric parameters (Å, º)

**Table:**

Tl1—O3^i^	2.867 (5)	Tl1—O1^vi^	3.216 (4)
Tl1—O3	2.889 (4)	P1—O2	1.469 (5)
Tl1—O3^ii^	2.935 (4)	P1—O3	1.497 (5)
Tl1—O2^iii^	2.943 (5)	P1—O1	1.595 (4)
Tl1—O2^iv^	2.963 (4)	P1—O1^vii^	1.606 (4)
Tl1—O2^v^	2.997 (4)		
			
O3^i^—Tl1—O3	77.73 (13)	Tl1^viii^—O1—Tl1^ix^	75.05 (8)
O3^i^—Tl1—O3^ii^	77.00 (13)	P1—O1—Tl1^x^	129.7 (2)
O3—Tl1—O3^ii^	93.54 (13)	P1^x^—O1—Tl1^x^	66.97 (15)
O3^i^—Tl1—O2^iii^	75.25 (13)	Tl1^viii^—O1—Tl1^x^	79.47 (8)
O3—Tl1—O2^iii^	109.83 (12)	Tl1^ix^—O1—Tl1^x^	73.65 (7)
O3^ii^—Tl1—O2^iii^	138.47 (13)	P1—O1—Tl1^v^	69.65 (16)
O3^i^—Tl1—O2^iv^	128.90 (12)	P1^x^—O1—Tl1^v^	130.4 (2)
O3—Tl1—O2^iv^	148.59 (14)	Tl1^viii^—O1—Tl1^v^	68.02 (8)
O3^ii^—Tl1—O2^iv^	79.42 (13)	Tl1^ix^—O1—Tl1^v^	79.10 (8)
O2^iii^—Tl1—O2^iv^	94.78 (12)	Tl1^x^—O1—Tl1^v^	141.92 (11)
O3^i^—Tl1—O2^v^	129.52 (12)	P1—O1—Tl1^xi^	61.69 (14)
O3—Tl1—O2^v^	79.59 (13)	P1^x^—O1—Tl1^xi^	68.71 (14)
O3^ii^—Tl1—O2^v^	149.02 (14)	Tl1^viii^—O1—Tl1^xi^	153.65 (12)
O2^iii^—Tl1—O2^v^	71.11 (16)	Tl1^ix^—O1—Tl1^xi^	131.30 (10)
O2^iv^—Tl1—O2^v^	90.79 (13)	Tl1^x^—O1—Tl1^xi^	105.89 (10)
O3^i^—Tl1—O1^vi^	47.18 (11)	Tl1^v^—O1—Tl1^xi^	112.06 (10)
O3—Tl1—O1^vi^	124.87 (13)	P1—O2—Tl1^ix^	115.5 (3)
O3^ii^—Tl1—O1^vi^	78.11 (12)	P1—O2—Tl1^iv^	148.1 (3)
O2^iii^—Tl1—O1^vi^	60.37 (11)	Tl1^ix^—O2—Tl1^iv^	85.22 (12)
O2^iv^—Tl1—O1^vi^	83.93 (12)	P1—O2—Tl1^v^	103.6 (2)
O2^v^—Tl1—O1^vi^	130.42 (13)	Tl1^ix^—O2—Tl1^v^	108.89 (16)
O3^i^—Tl1—P1	87.23 (9)	Tl1^iv^—O2—Tl1^v^	90.79 (13)
O3—Tl1—P1	24.76 (9)	P1—O2—Tl1	74.9 (2)
O3^ii^—Tl1—P1	73.32 (9)	Tl1^ix^—O2—Tl1	149.54 (14)
O2^iii^—Tl1—P1	134.59 (9)	Tl1^iv^—O2—Tl1	75.68 (11)
O2^iv^—Tl1—P1	127.54 (11)	Tl1^v^—O2—Tl1	95.07 (13)
O2^v^—Tl1—P1	90.46 (10)	P1—O2—Tl1^viii^	82.93 (19)
O1^vi^—Tl1—P1	130.72 (8)	Tl1^ix^—O2—Tl1^viii^	66.99 (9)
O3^i^—Tl1—O2	108.64 (12)	Tl1^iv^—O2—Tl1^viii^	128.67 (15)
O3—Tl1—O2	45.47 (11)	Tl1^v^—O2—Tl1^viii^	61.83 (8)
O3^ii^—Tl1—O2	69.49 (11)	Tl1—O2—Tl1^viii^	143.17 (12)
O2^iii^—Tl1—O2	149.54 (14)	P1—O3—Tl1^xi^	107.8 (3)
O2^iv^—Tl1—O2	104.32 (11)	P1—O3—Tl1	101.3 (2)
O2^v^—Tl1—O2	84.93 (13)	Tl1^xi^—O3—Tl1	103.19 (15)
O1^vi^—Tl1—O2	144.09 (10)	P1—O3—Tl1^xii^	142.3 (3)
P1—Tl1—O2	23.87 (8)	Tl1^xi^—O3—Tl1^xii^	102.06 (14)
O2—P1—O3	121.3 (3)	Tl1—O3—Tl1^xii^	93.54 (13)
O2—P1—O1	105.7 (3)	P1—O3—Tl1^v^	72.3 (2)
O3—P1—O1	110.3 (2)	Tl1^xi^—O3—Tl1^v^	163.77 (15)
O2—P1—O1^vii^	110.2 (2)	Tl1—O3—Tl1^v^	92.54 (13)
O3—P1—O1^vii^	104.6 (3)	Tl1^xii^—O3—Tl1^v^	72.64 (11)
O1—P1—O1^vii^	103.41 (12)		

Symmetry codes: (i) −*x*+1/2, *y*−1/2, −*z*+3/2; (ii) *x*, *y*−1, *z*; (iii) *x*, *y*, *z*+1; (iv) −*x*, −*y*, −*z*+1; (v) −*x*, −*y*+1, −*z*+1; (vi) *x*, *y*−1, *z*+1; (vii) −*x*+1/2, *y*−1/2, −*z*+1/2; (viii) *x*, *y*+1, *z*−1; (ix) *x*, *y*, *z*−1; (x) −*x*+1/2, *y*+1/2, −*z*+1/2; (xi) −*x*+1/2, *y*+1/2, −*z*+3/2; (xii) *x*, *y*+1, *z*.
